# Burnout syndrome and the protective role of resilience among university professors: a cross-sectional study of associated psychosocial and physical factors

**DOI:** 10.3389/fpsyg.2025.1616489

**Published:** 2025-08-01

**Authors:** Maria Jose Alban Guijarro, Luis Chauca-Bajaña, Carlos Martínez Florencia, José Dionel Alban Sanchez, Miguel Álvarez Aviles, Denisse Arroyo-Apolo, Andrea Ordoñez Balladares, Manuel Roberto Tolozano-Benites, Alba Perez-Jardon, Byron Velásquez Ron

**Affiliations:** ^1^University of Guayaquil, Guayaquil, Ecuador; ^2^Periodontics and Implantology Oral Research, College Dentistry, University of Guayaquil, Guayaquil, Ecuador; ^3^Especialização em Dentistica, College of Dentistry, University of Guayaquil, Guayaquil, Ecuador; ^4^Faculty of Dentistry, Universidad Bolivariana del Ecuador, Durán, Ecuador; ^5^Universidad Bolivariana del Ecuador, Durán, Ecuador; ^6^Oral Medicine, Oral Surgery and Implantology Unit (MedOralRes), Faculty of Medicine and Dentistry, Universidade de Santiago de Compostela, Health Research Institute of Santiago de Compostela (IDIS), (ORALRES GROUP), Santiago de Compostela, A Coruña, Spain; ^7^Dental Prosthesis Department Research, College Dentistry, Universidad de Las Americas (UDLA), Quito, Ecuador

**Keywords:** resilience, burnout, professor, symptom, stress, education, psychology, exhaustion

## Abstract

**Background:**

Burnout syndrome is increasingly common among university professors, who face demanding workloads, academic pressures, and challenging work environments. This condition negatively impacts not only their emotional and physical well-being but also the quality of education. This study aimed to determine the prevalence of burnout syndrome and its associations with physical symptoms, resilience, and sociodemographic variables among professors at the University of Guayaquil.

**Methods:**

A cross-sectional study was conducted with a random sample of 334 university professors. Three instruments were used: the Maslach Burnout Inventory (MBI), a physical symptomatology questionnaire, and the Connor-Davidson Resilience Scale. Data analysis was performed using SPSS version 27. Chi-square tests were applied to identify associations between variables, and simple linear regression analysis was used to explore predictors of burnout, particularly the role of resilience and physical symptoms.

**Results:**

Of the professors surveyed, 52.4% were men and 47.6% women. Emotional exhaustion had a mean of 22.05 (SD = 5.665), and depersonalization had a mean of 8.37 (SD = 3.092). Physical symptoms were reported by 71.6% of participants, with nausea (86.2%) and respiratory issues (81.1%) being the most prevalent. Regression analysis revealed that employment status and several physical symptoms (e.g., sleep disturbances, muscle tension, gastrointestinal discomfort) were significantly associated with higher burnout levels (*p* < 0.05). Resilience was inversely associated with burnout, confirming its protective role (*p* < 0.01).

**Conclusion:**

A significant proportion of university professors experience moderate to high levels of burnout, especially emotional exhaustion and depersonalization. Resilience was identified as a key protective factor, emphasizing the importance of institutional strategies that promote psychological well-being and support among faculty members.

## 1 Introduction

Burnout Syndrome (BS) was first described in Freudenberger (1974) (Chauca Bajaña et al., 2023). It is considered one of the main health disorders directly linked to the work environment (Wisniewska and Richard Holt, 2023). In this context, special attention to the mental well-being of professors is crucial (Weiskopf, 1980; Deroncele-Acosta et al., 2023). Maslach describes it in three interrelated dimensions: emotional exhaustion, depersonalization, and lack of personal accomplishment (Maslach and Leiter, 2016; Maslach and Leiter, 2008; Portoghese et al., 2018; Dres et al., 2023). Burnout Syndrome is a psychosocial phenomenon that arises in response to chronic interpersonal stressors (Moch et al., 2003). It is estimated that BS affects between 41% and 68% of university professors (Silva et al., 2021; Moueleu Ngalagou et al., 2019), manifesting in a wide range of symptoms, from emotional aspects to psychiatric, cognitive, and psychosomatic manifestations, with varying degrees of severity (Bauer et al., 2006; Ogińska-Bulik and Juczyński, 2021; Alqassim et al., 2022). The emergence of the COVID-19 pandemic introduced new stressors for professors, who had to adapt their work methods, incorporating Information and Communication Technologies (ICT) into both pedagogy and academic content (Altwaim et al., 2023; Prado-Gascó et al., 2020; Price, 2020). A systematic review revealed that the pandemic significantly impacted mental health, increasing stress, anxiety, and temporomandibular disorders (TMD), with headaches being a common manifestation (Minervini et al., 2023). Professors often face stress due to factors such as managing student and parent behavior, the diversity of students with their communication demands, conflicting role expectations, high social interaction (Beutel et al., 2023), and the lack of time to balance their professional responsibilities, continuous training, and recreational activities (Leite et al., 2019). The well-being of educators directly impacts the quality of teaching and, therefore, influences the success of the learning process (Scheuch et al., 2015). Preventing job burnout involves improving working conditions, balancing job responsibilities, and regularly engaging in physical activities (Ahola et al., 2012).

Resilience is the ability to negotiate, adapt, and effectively manage significant sources of stress or trauma (Windle, 2011). Among university professors, resilience refers to the ability to face and overcome the challenges inherent in their work, maintaining their well-being and teaching effectiveness (Mansfield et al., 2016; Gu and Day, 2007). Currently, various scales and questionnaires exist to measure resilience, such as the Resilience Scale (RS) (Wagnild and Young, 1993), Ego-Resilience (Block and Kremen, 1996), Resilience Attitudes and Skills Profile (Hurtes and Allen, 2001), Connor-Davidson Resilience Scale (CD-RISC) (Connor and Davidson, 2003), Adolescent Resilience Scale (Oshio et al., 2003), Adult Resilience Scale (Friborg et al., 2005; Friborg et al., 2003), Dispositional Resilience Scale (Bartone, 2007), 10-Item Connor-Davidson Resilience Scale (Campbell-Sills and Stein, 2007), Youth Resilience: Assessing Developmental Strengths (YR: ADS) (Donnon and Hammond, 2007), California Healthy Kids Survey Resilience Scale (Sun and Stewart, 2007), Brief Resilience Scale (Smith et al., 2008), Child and Youth Resilience Measure (CYRM) (Ungar et al., 2008), and the Psychological Resilience Scale (Windle et al., 2008).

The objective of this study is to determine the prevalence of burnout syndrome and associated factors among professors at the University of Guayaquil.

## 2 Materials and methods

### 2.1 Study design and data collection

This study presents an exploratory, analytical, correlational, and cross-sectional approach, conducted with professors from various faculties at the University of Guayaquil during the second semester of 2023–2024. An authorization letter was sent to each dean explaining the application of the data collection instrument and the research objectives. The study has the approval of the 2023 Research Project at the University of Guayaquil, identified by code FCI-049-2023 and the Ethics Committee of the Universidad de las Américas (Ethical Committee CBE/UDLA17052408). It adheres to the ethical principles established in the Declaration of Helsinki. Informed consent to participate was obtained from all participants prior to their inclusion in the study. Participants were thoroughly informed about the research objectives, procedures, potential risks, and benefits. They were assured that their participation was voluntary and that they could withdraw from the study at any time without any negative consequences. All collected data were treated confidentially and used solely for research purposes, ensuring compliance with ethical and legal standards. The issue of missing data and non-responses was handled by excluding incomplete questionnaires from the final analysis.

### 2.2 Participants and sample calculation

The total population consisted of 2,515 active professors. The required sample size was calculated using the formula for simple random sampling in finite populations, with a 95% confidence level (Z = 1.96), an assumed response distribution of 50%, and a margin of error of 5%. This resulted in a minimum sample size of 334 professors. To ensure randomness, a list of all professors was obtained from the university’s human resources database, and random selection was carried out using a computerized random number generator. Each selected professor received a digital invitation via institutional email, which included a link to the online survey along with a brief explanation of the study’s purpose. One reminder was sent after 1 week to increase participation. Participation was voluntary, anonymous, and entirely conducted through a secure online platform.

### 2.3 Research instrument

The instrument consists of three questionnaires. First, the Maslach Burnout Inventory (MBI) is used to assess the attitudes and feelings of professionals toward their work (García-Real et al., 2024; Marić et al., 2022). The inventory is applied in its educational form to measure the level of burnout experienced by university professors. It consists of 22 items divided into three categories: Emotional exhaustion, made up of 9 questions (1, 2, 3, 6, 8, 13, 14, 16, and 20), which estimate the level of emotional fatigue perceived by the professor (Sánchez-Pujalte et al., 2023; Kalamara and Richardson, 2022; Seibt and Kreuzfeld, 2021). Depersonalization consists of 5 items (5, 10, 11, and 22), evaluating the degree to which the professor recognizes feelings of detachment and coldness toward their work (Abdelmounaim et al., 2022; Masluk et al., 2022; Neto et al., 2023). Personal accomplishment is assessed with 8 items (4, 7, 9, 12, 17, 18, 19, and 21), which evaluate the professor’s sense of personal achievement (Martínez et al., 2020; Taylor et al., 2021). Each statement is rated on a six-point Likert scale: Never = 0, Almost never = 1, Sometimes = 2, Often = 3, Frequently = 4, Very often = 5, Every day = 6. An exception is made for the Personal Accomplishment dimension, where the scale is reversed. In studies on professional burnout, high scores in emotional exhaustion and depersonalization, combined with low scores in personal accomplishment, define the syndrome (Juárez-García et al., 2023). For the tabulation of results, scores of 0 to 18 are considered low, 19 to 26 medium, and 27 to 54 high for emotional exhaustion. For depersonalization, 0 to 5 is low, 6 to 9 is medium, and 10 to 30 is high. Finally, for personal accomplishment, scores from 0 to 33 are low, 34 to 39 are medium, and 40 to 56 are high in burnout syndrome (Sánchez-Pujalte et al., 2023; Martínez et al., 2020).

The second questionnaire explores symptoms related to professional burnout, including stomach aches, fatigue, sleep problems, respiratory issues, and musculoskeletal discomfort (Chauca Bajaña et al., 2023). It contains 14 items, each rated on a five-point Likert scale: Never (1), Almost never (2), Regularly (3), Sometimes (4), Frequently (5). The purpose of this questionnaire is to assess the frequency of these symptoms and their relationship to the levels of burnout found in the study population. The instrument was developed based on previously documented physical manifestations of burnout in academic settings (Bauer et al., 2006; Chauca Bajaña et al., 2023). To evaluate internal consistency, we calculated Cronbach’s alpha, obtaining a value of 0.827, which reflects good reliability.

The Connor-Davidson Resilience Scale (CD-RISC) (Faria Anjos et al., 2019; Kuiper et al., 2019) measures the professor’s ability to adapt to the challenging situations encountered in their teaching work (Xie, 2021; Nituica et al., 2021a; Padmanabhanunni et al., 2023). It consists of 25 items, where the professor indicates the extent to which each statement reflects their perception of their work with students, on a scale of 0 = Not at all, 1 = Rarely, 2 = Sometimes, 3 = Often, 4 = Almost always (Chu and Liu, 2022). Total scores range from 0 to 100, with higher scores indicating greater resilience (Papini et al., 2021).

### 2.4 Statistical analysis

The data collected for this study is quantitative and are presented in frequency and percentage tables, as well as descriptive tables that include central position statistics such as mean, median, standard deviation, and variance. For relational tests, the Chi-Square test was used to determine the significance level between two variables. Simple linear regression analysis was applied to examine the relationships between the total burnout score (independent variable) and the subdimensions: emotional exhaustion, depersonalization, and personal accomplishment. Regression coefficients, confidence intervals, and R^2^ values were calculated. A *p*-value < 0.05 was considered statistically significant. Given the exploratory nature of this study and the objective to identify possible psychosomatic and psychosocial correlates of burnout, we performed multiple chi-square analyses. Although no correction for multiple comparisons was applied, the results were interpreted with caution and emphasis was placed on patterns that were theoretically supported and consistent across symptom categories. All data processing and result analysis were conducted using SPSS statistical software, version 27.

## 3 Results

The sample in this study included 334 university professors, with a gender distribution: 52.4% were men and 47.6% were women. Analyzing the internal consistency of the instruments used, we found values demonstrating good reliability: Cronbach’s alpha for the burnout questionnaire was 0.868, indicating strong performance in measurement. The symptomatology questionnaire had an alpha of 0.827, while the resilience questionnaire achieved 0.957, showing excellent item correlation.

The results reveal that emotional exhaustion and depersonalization are significant issues for many participants, while the perception of personal accomplishment is relatively low (Table 1).

**Table 1:** Prevalence of burnout syndrome by dimensions.

	Low		Moderate		High	
	*n*	%	*n*	%	*n*	%
Emotional exhaustion	98	29.3	172	51.5	64	19.2
Depersonalization	27	8.1	210	62.9	97	29.0
Personal fulfillment	333	99.7	1	0.3	0	0.0

In terms of emotional exhaustion, professors were at a moderate level, with a mean of 22.05 (SD = 5.665), and depersonalization, although less intense, remained relevant, with a mean of 8.37 (SD = 3.092). Regarding personal accomplishment, participants reported low levels, with a mean score of 22.91 (SD = 3.370). Since this dimension is inversely scored in the MBI, lower values indicate greater burnout severity. Therefore, this result reflects a high level of burnout in this domain (Table 2).

**Table 2:** Descriptive analysis of burnout syndrome.

Dimensions	Statistical ratings					
	Min	Max	Mean	Standard error	SD	CI
Emotional exhaustion	8.0	43.0	22.1	0.3	5.7	21.4–22.7
Depersonalization	3.0	21.0	8.4	0.2	3.1	8.0–8.7
Personal fulfillment	9.0	34.0	22.9	0.2	3.4	22.6–23.3

**Figure 1:**
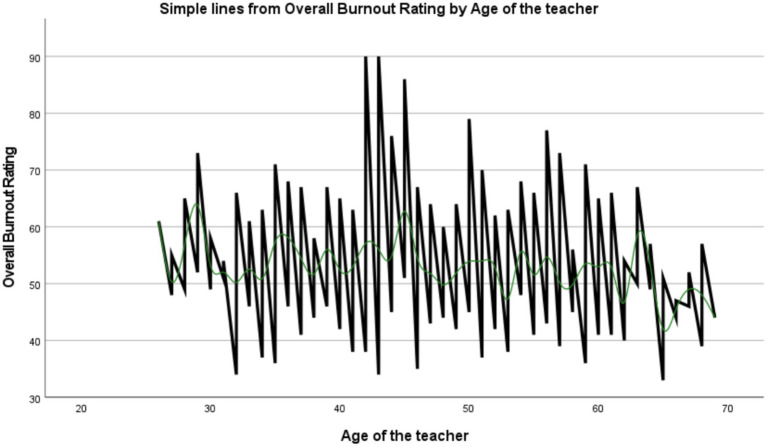
Relationship between teacher age and overall burnout rating.

The results showed a progressive decrease in burnout levels with increasing age among teachers, and this trend stabilized notably after the age of 50. Younger teachers exhibited higher and more variable levels of burnout, indicating a greater susceptibility to work-related stress in the early stages of their careers. In contrast, older teachers experienced more moderate and consistent levels of burnout (Figure 1).

The relationship between physical and emotional well-being and discomfort in professors was found to be complex. Although 69.5% felt capable of making quick decisions and 52.1% enjoyed recreational activities, high levels of physical discomfort were also reported. This included symptoms such as sleep difficulties (47.3%), muscle tension (47.3%), and pain in areas like the head (51.5%), neck (41.9%), and temporomandibular joint (72.8%). These findings were complemented by the prevalence of other symptoms, such as appetite loss (71.6%), nausea or vomiting (86.2%), and respiratory issues (81.1%), suggesting widespread discomfort. Additionally, physical symptoms related to stress, such as hand or eyelid tremors (72.2%) and sweaty or cold hands (60.5%), were observed (Table 3).

**Table 3:** Distribution of symptoms.

	Never		Almost never		Regularly		Sometimes		Many times	
	*n*	%	*n*	%	*n*	%	*n*	%	*n*	%
Sleeping issues	158	47.3	142	42.5	34	10.2	0	0.0	0	0.0
Tension	146	43.7	111	33.2	35	10.5	35	10.5	7	2.1
Quick decisions	4	1.2	7	2.1	32	9.6	59	17.7	232	69.5
Enjoying activities	0	0.0	16	4.8	54	16.2	90	26.9	174	52.1
Headaches	172	51.5	100	29.9	21	6.3	35	10.5	6	1.8
Neck pain	140	41.9	92	27.5	48	14.4	38	11.4	16	4.8
ATM pain	243	72.8	55	16.5	14	4.2	17	5.1	5	1.5
Back and waist pain	122	36.5	97	29.0	46	13.8	49	14.7	20	6.0
Pain in the body extremities	171	51.2	73	21.9	38	11.4	41	12.3	11	3.3
Stomach or digestive pain/discomfort	173	51.8	106	31.7	21	6.3	24	7.2	10	3.0
Easily fatigue	122	36.5	124	37.1	29	8.7	46	13.8	13	3.9
Loss of appetite	239	71.6	72	21.6	13	3.9	8	2.4	2	0.6
Increase of appetite	137	41.0	96	28.7	41	12.3	48	14.4	12	3.6
Nausea and vomit	288	86.2	31	9.3	9	2.7	5	1.5	1	0.3
Shaky hands	241	72.2	62	18.6	12	3.6	15	4.5	4	1.2
Cold or sweaty hands	202	60.5	34	10.2	61	18.3	29	8.7	8	2.4
Respiratory issues	271	81.1	45	13.5	6	1.8	11	3.3	1	0.3
Menstrual cycle is irregular	88	55.3	23	14.5	16	10.1	12	7.5	20	12.6

Professors demonstrated notable resilience in aspects such as adapting to change, maintaining close relationships, and confidence in facing new challenges. However, the presence of physical and emotional symptoms associated with stress, such as nausea, tremors, and respiratory issues, indicates that some could benefit from interventions that strengthen their resilience against stress (Table 4).

**Table 4:** Distribution of resilience.

Dimension	High resilience (%)	Moderate (%)	Low (%)
Adaptation to changes	60.8	19.8	15.0
Close and secure relationships	69.8	7.8	20.1
Destiny or god can help	50.0	14.7	18.3
Facing whatever comes	59.0	19.8	15.9
Confidence for new challenges	63.5	11.7	18.0
The fun side of things	48.2	28.1	12.9
Stress strengthens	41.9	24.9	15.3
Illness or a difficulty	56.9	21.0	15.9
Things happen for a reason	47.0	23.4	14.1
I give my best	70.4	7.2	20.7
Achieving your goals	65.9	15.9	16.8
I do not give up	52.1	11.1	27.5
Turn to seek help	53.3	22.2	16.5
I think clearly	47.6	24.9	14.4
Problem solving	49.1	24.9	14.1
I do not get discouraged by failures	40.7	18.9	22.5
Strong person	56.0	20.1	16.5
Unpopular or difficult decisions	24.0	21.0	12.9
Unpleasant feelings	41.9	27.8	12.6
Acting on instincts	4.2	8.1	19.2
They have meaning	59.9	20.1	17.7
Control of my life	57.5	20.1	20.1
I like challenges	56.3	17.4	16.5
You work to achieve your goals	67.7	12.0	19.5
I am proud of my achievements	70.4	6.3	21.0

Regarding demographic and occupational characteristics, no statistically significant association was found between burnout and variables such as gender, marital status, age, years of experience, and time working at the institution (*p* > 0.05). However, employment status showed a significant relationship with burnout (*p* = 0.049). Additionally, physical and emotional stress symptoms, such as sleep problems, muscle tension, physical pain, and gastrointestinal disorders, were strongly associated with burnout (*p* < 0.001). Resilience factors, such as the ability to adapt to change, achieving goals, clarity under pressure, and persistence in the face of difficulties, were also significantly related to burnout (*p* < 0.01) (Table 5).

**Table 5:** Prevalence of burnout syndrome in relation to teacher characteristics, teaching characteristics, symptoms, and resilience.

Group	Variable	Burnout		
		Chi^2^	gl	*p*
Teacher characteristics	Sex	0.599	1	0.439
	Marital status	2.033	5	0.845
	Age	35.810	43	0.773
	Years of experience	22.893	40	0.986
	Years working in the institution	34.782	34	0.431
Teaching characteristics	Faculty in which they primarily teach classes	20.505	16	0.198
	Work schedule	0.669	2	0.716
	Employment situation*	3.610	1	0.049
	Highest academic level achieved	8.065	4	0.089
	Extracurricular activity	9.334	7	0.230
	Job category	2.038	1	0.153
	Semesters in which they teach classes	3.612	2	0.164
Symptoms	Sleeping issues*	25.978	2	0.000
	Tension*	55.700	4	0.000
	Quick decisions*	18.260	4	0.001
	Enjoying activities*	30.274	3	0.000
	Headaches*	26.823	4	0.000
	Neck pain*	32.675	4	0.000
	ATM pain*	25.000	4	0.000
	Back and waist pain*	37.419	4	0.000
	Pain in the body extremities*	43.012	4	0.000
	Stomach or digestive pain/discomfort*	24.273	4	0.000
	Easily fatigue*	46.678	4	0.000
	Loss of appetite*	19.786	4	0.001
	Increase of appetite*	24.326	4	0.000
	Nausea and vomit*	20.668	4	0.000
	Trembling in the hands or eyelids*	35.748	4	0.000
	Cold or sweaty hands*	11.784	4	0.019
	Respiratory issues	8.255	4	0.083
	Menstrual cycle is irregular	2.201	4	0.699
Resilience	Adaptation to changes*	17.976	4	0.001
	Close and secure relationships	8.909	4	0.063
	Destiny or god can help	6.540	4	0.162
	Facing whatever comes	6.189	4	0.185
	Confidence for new challenges	7.114	4	0.130
	The fun side of things	4.744	4	0.315
	Stress strengthens	7.928	4	0.094
	Illness or a difficulty	5.386	4	0.250
	Things happen for a reason	4.717	4	0.318
	I give my best	6.291	4	0.178
	Achieving your goals*	15.960	4	0.001
	I do not give up	8.787	4	0.067
	Turn to seek help	8.736	4	0.068
	I think clearly*	16.125	4	0.003
	Problem solving	3.141	4	0.535
	I do not get discouraged by failures*	19.185	4	0.001
	Strong person	5.769	4	0.217
	Unpopular or difficult decisions	7.510	4	0.111
	Unpleasant feelings	5.126	4	0.275
	Acting on instincts	6.956	4	0.138
	They have meaning	6.362	4	0.174
	Control of my life	4.678	4	0.197
	I like challenges*	10.353	4	0.035
	You work to achieve your goals	4.092	4	0.252
	I am proud of my achievements*	14.910	4	0.002

*The chi-square statistic is significant at the 0.05 level.

The linear regression models and corresponding scatter plots illustrated positive relationships between the total burnout score and its subdimensions: emotional exhaustion, depersonalization, and personal accomplishment, although at varying levels of intensity.

**Figure 2:**
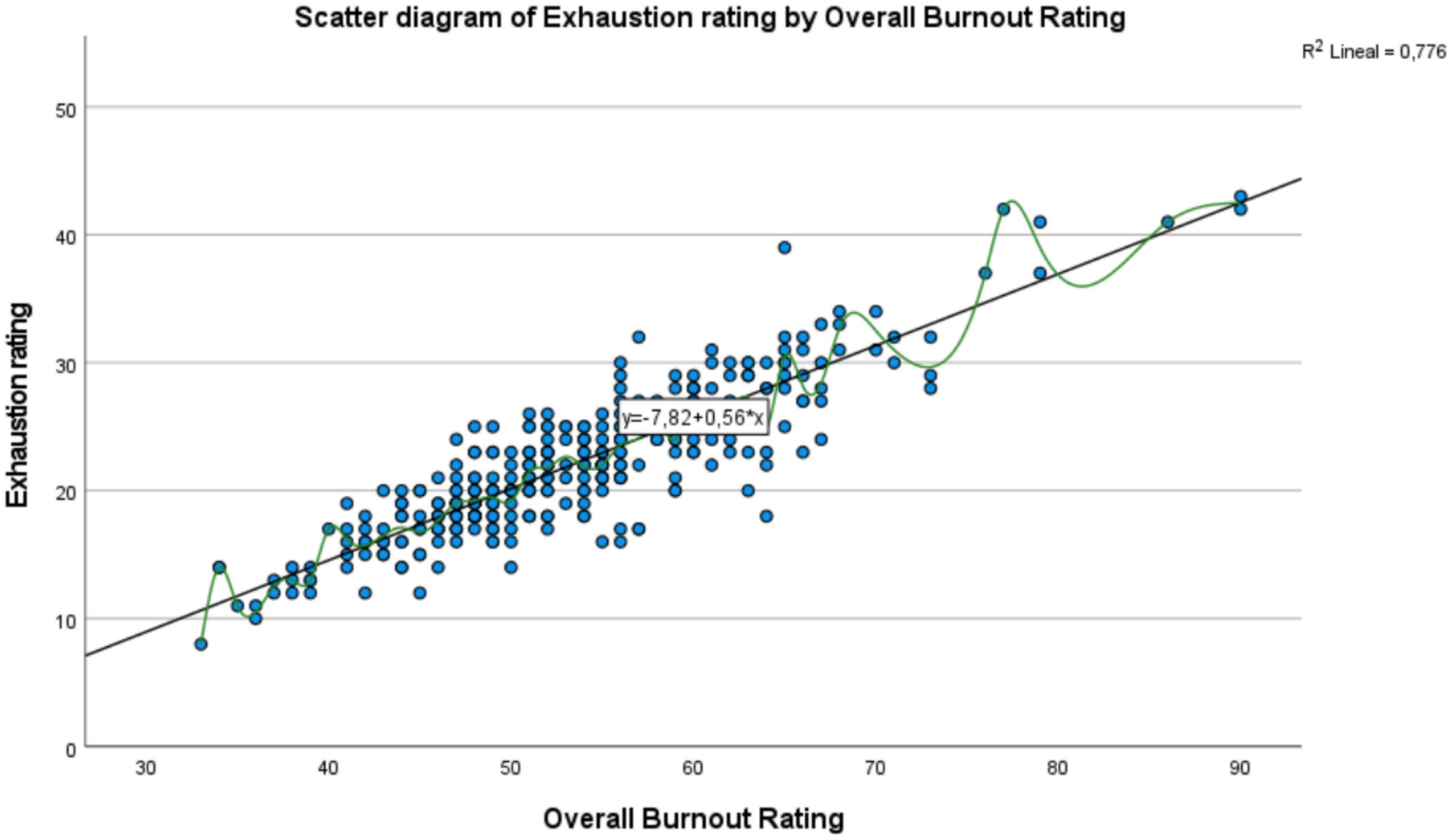
Scatter diagram: relationship between two variables: total burnout score (x-axis) and exhaustion score (y-axis).

**Figure 3:**
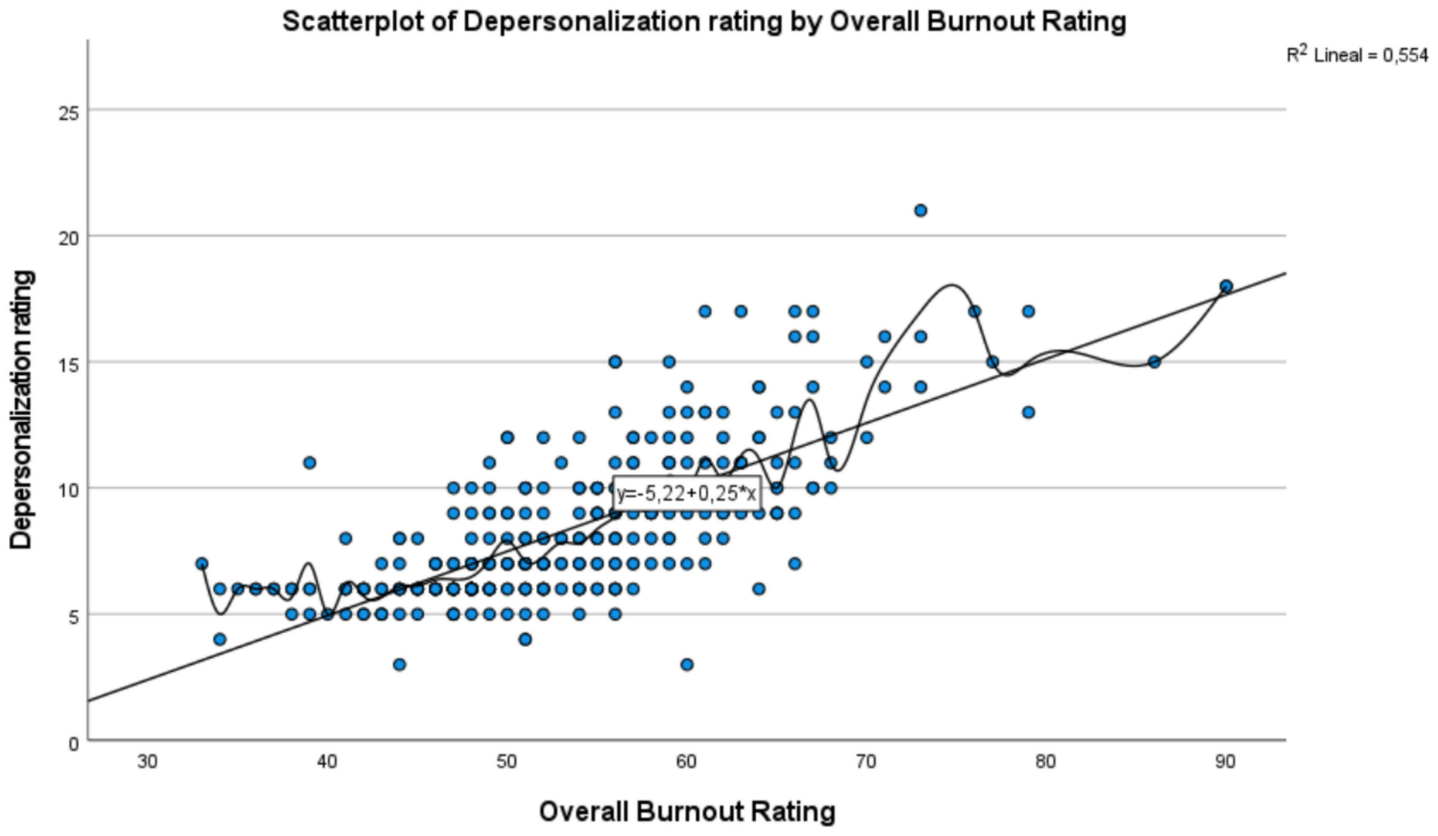
Scatter diagram: relationship between total burnout score (x-axis) and depersonalization score (y-axis).

**Figure 4:**
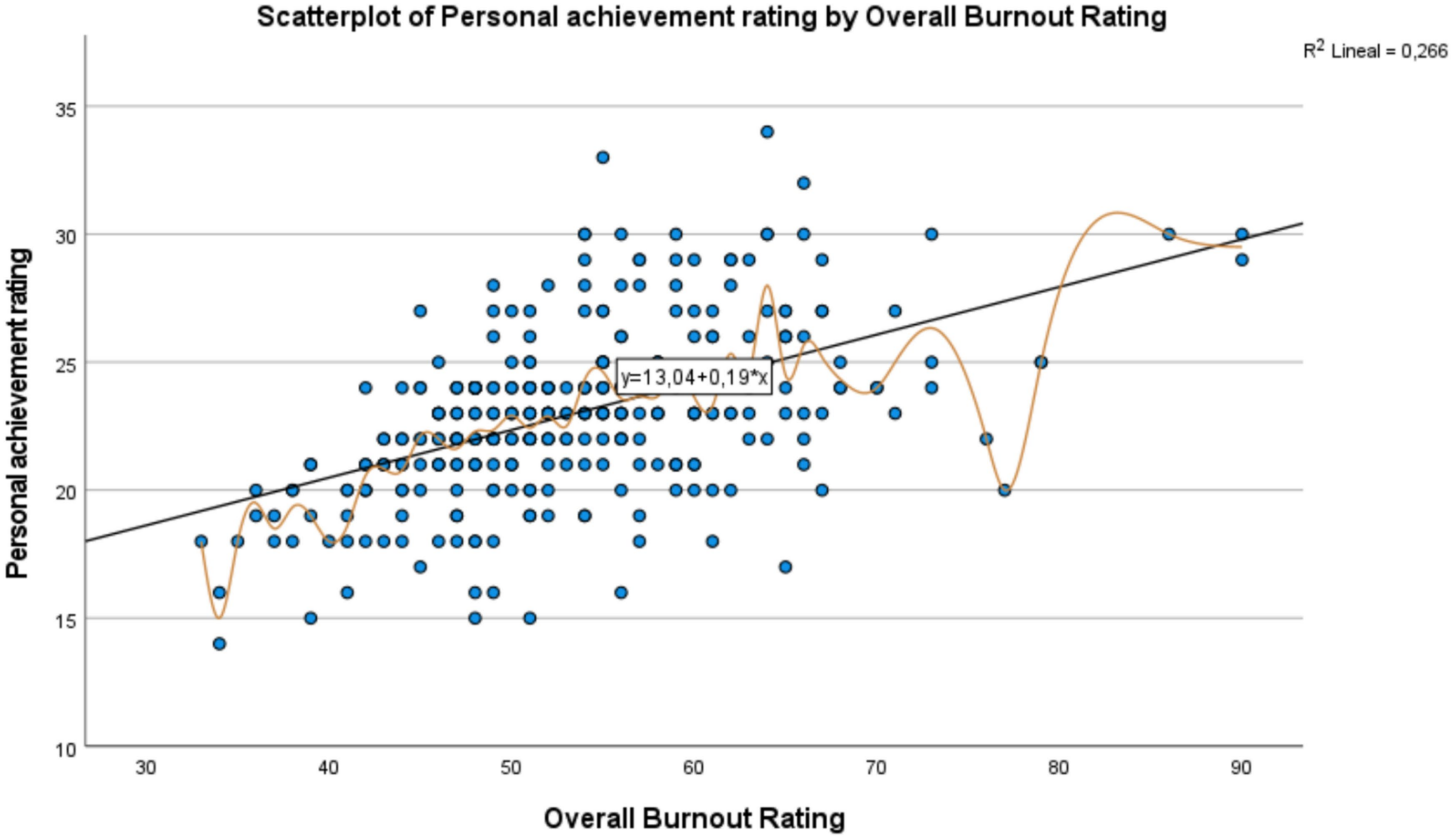
Scatter diagram: relationship between total burnout score (x-axis) and personal accomplishment score (y-axis).

In Figure 2, which shows the relationship between the Total Burnout Score and the Exhaustion Score, a positive trend is observed: as burnout increases, exhaustion also increases, suggesting a positive correlation between the two variables. The regression line, represented by the equation y = −7.82 + 0.56𝑥, indicates that each additional point in burnout increases exhaustion by an average of 0.56 points. With a coefficient of determination of R2 = 0.776 it is concluded that 77.6% of the variability in exhaustion is explained by burnout, reflecting a strong relationship. Despite the clear trend, the spread of some points suggests the influence of other factors or individual variability in exhaustion. In Figure 3, a moderate positive relationship is observed between the Total Burnout Score and the Depersonalization Score: as burnout increases, depersonalization tends to increase as well, though this relationship is less consistent than with exhaustion. The trendline equation y = −5.22 + 0.25x suggests that, on average, each additional point in burnout increases depersonalization by 0.25 points. However, the coefficient of determination R2 = 0.554 indicates that 55.4% of the variability in depersonalization is explained by burnout, suggesting the possible influence of other factors or greater individual variability in this dimension. In Figure 4, which shows the relationship between the Total Burnout Score and the Personal Accomplishment Score, a positive but weak trend is observed. Although an increase in burnout is associated with a slight increase in personal accomplishment, the relationship is not as significant as in the cases of exhaustion and depersonalization. The trendline equation is y = 13.04 + 0.19𝑥, indicating that each additional point in burnout increases personal accomplishment by only 0.19 points on average. With a coefficient of determination of R2 = 0.266, only 26.6% of the variability in personal accomplishment is explained by burnout, suggesting a weak relationship and the possible influence of other factors. Additionally, the wide spread of points around the regression line reinforces that this relationship is variable and less predictable.

## 4 Discussion

University professors are increasingly exposed to emotional and physical demands that can erode their well-being over time. The literature has consistently documented high rates of emotional exhaustion and depersonalization in this population. In line with these findings, 51.5% of the professors in the present sample reported moderate levels of emotional exhaustion, while 62.9% experienced moderate depersonalization. However, what stands out most is the overwhelming sense of diminished personal accomplishment: 99.7% of participants reported low scores in this dimension. This suggests that while many professors are navigating ongoing emotional and interpersonal strain, an even greater number are struggling with a deeper sense of disengagement and loss of meaning in their professional roles an aspect that may have more lasting consequences for both educators and the quality of education they provide. These findings are consistent with the study by Teles et al. (2020), which analyzed 520 higher education professors and found significant levels of emotional exhaustion. In that study, 31.3% of the variance in burnout was attributed to perceived stress, highlighting the importance of considering this factor when studying the syndrome. Similarly, Agyapong et al. (2024) research revealed that among 1,912 professors, 76.9% experienced emotional exhaustion, 23.2% depersonalization, and 30.8% lack of personal accomplishment. This shows that burnout syndrome is becoming a growing issue in the academic context.

The participants displayed high levels of resilience. For example, 70.4% stated: “I give my best,” and another 70.4% said, “I am proud of my achievements.” Additionally, 69.8% reported having “close and secure relationships.” These results suggest that resilience may be a key protective factor, helping many professors avoid high levels of burnout in terms of emotional exhaustion and depersonalization. In the dimension of personal accomplishment, resilience seems to have a less marked relationship with the prevention of severe derealization. This indicates that resilience, understood as the ability to adapt to changes and face challenges positively, has a favorable influence on burnout syndrome. Therefore, higher levels of resilience correspond to lower levels of emotional exhaustion and depersonalization experienced by university professors (Nituica et al., 2021b).

Thus, it can be concluded that resilience acts as a protective factor against burnout (Agyapong et al., 2023). This research suggests that fostering resilience can be a valuable tool in preventing the effects of the syndrome, being an intrinsic skill that allows professors to cope with the demands of the academic environment and achieve their goals (Melguizo-Ibáñez et al., 2023). In this context, it is crucial to implement strategies that strengthen professors’ motivation in their academic activities, such as creating a recognition system for their achievements (Salmela-Aro et al., 2019) or organizing events that promote positive dynamics among colleagues. It would also be beneficial to carry out continuous evaluations of professors’ perceptions of their own effectiveness (Nituica et al., 2021a; Lebares et al., 2018). In this way, proactive support can be provided in their teaching work to prevent burnout. Additionally, a study by Melguizo-Ibáñez et al. (2022) with 4,117 professors found that those who practiced at least 3 h of physical activity per week showed a better association between resilience, burnout, and stress. They concluded that physical activity can help reduce stress and burnout syndrome, thus mitigating its effects on professors’ physical health. These findings align with the physical symptoms observed in our research. Regarding the physical symptoms accompanying burnout, common issues were identified, such as sleep difficulties (47.3%), tension (47.3%), and pain in areas like the head (51.5%), neck (41.9%), and temporomandibular joint (72.8%). Furthermore, a high prevalence of symptoms such as appetite loss (71.6%), nausea or vomiting (86.2%), and respiratory problems (81.1%) were reported. This suggests that burnout syndrome can generate a wide range of physical ailments in professors that, if not addressed promptly, may worsen their health. Therefore, greater involvement of physical therapists in the work environment is recommended (Quinn et al., 2021).

It is unsurprising that many educational institutions are implementing active breaks during the workday, allowing professors to engage in short, dynamic exercises that make the day more manageable (Galof and Šuc, 2021). Additionally, higher education institutions should ensure that professors have access to health plans that allow for quick and effective treatment of their physical ailments (Bogaert et al., 2014). Moreover, investing in educational campaigns about physical and mental health would be valuable, helping professors recognize the negative effects that emotional exhaustion and depersonalization can have on their physical well-being so they can take appropriate steps and seek timely medical attention (Kotowski et al., 2022). Finally, although this study focused on exploring resilience and physical symptoms associated with burnout, it is clear that much remains to be understood. Investigating the factors that influence academic burnout is essential—not only to identify protective and risk variables, but also to support the well-being of educators and strengthen the learning environment as a whole. When a professor feels motivated, resilient, and fulfilled in their role, it is more likely that their students will also benefit, both emotionally and academically. It is important to acknowledge that our research was conducted within a single public university in Ecuador. Institutional characteristics such as workload, administrative duties, and academic culture—can shape how burnout manifests and is reported. As a result, the findings may not fully reflect the experience of faculty in other educational settings. We therefore recommend that future studies adopt multicenter approaches that include diverse institutions, allowing for broader and more representative insights. Interestingly, we observed a tendency toward lower burnout levels among professors aged 50 and older. While this finding is noteworthy, it should be interpreted with caution, as our analysis did not control related factors such as teaching seniority, job security, or potential reductions in teaching load due to administrative roles. Future research using multivariate models or ANOVA is needed to better understand the independent influence of age on burnout in academic professionals.

## 5 Conclusion

A significant proportion of professors at the University of Guayaquil experience burnout syndrome, especially in the dimensions of emotional exhaustion and depersonalization. However, resilience was identified as a key protective factor, suggesting the need to implement institutional strategies that strengthen this capacity to reduce the impact of burnout and improve professors’ well-being.

## Data Availability

The original contributions presented in the study are included in the article/supplementary material, further inquiries can be directed to the corresponding author.
